# Evaluation of a Web-Based App Demonstrating an Exclusionary Algorithmic Approach to TNM Cancer Staging

**DOI:** 10.2196/cancer.4019

**Published:** 2015-04-02

**Authors:** Matthew Kim

**Affiliations:** ^1^ Brigham and Women's Hospital Division of Endocrinology, Diabetes and Hypertension Boston, MA United States

**Keywords:** TNM staging, neoplasms, medical oncology, instructional technology

## Abstract

**Background:**

TNM staging plays a critical role in the evaluation and management of a range of different types of cancers. The conventional combinatorial approach to the determination of an anatomic stage relies on the identification of distinct tumor (T), node (N), and metastasis (M) classifications to generate a TNM grouping. This process is inherently inefficient due to the need for scrupulous review of the criteria specified for each classification to ensure accurate assignment. An exclusionary approach to TNM staging based on sequential constraint of options may serve to minimize the number of classifications that need to be reviewed to accurately determine an anatomic stage.

**Objective:**

Our aim was to evaluate the usability and utility of a Web-based app configured to demonstrate an exclusionary approach to TNM staging.

**Methods:**

Internal medicine residents, surgery residents, and oncology fellows engaged in clinical training were asked to evaluate a Web-based app developed as an instructional aid incorporating (1) an exclusionary algorithm that polls tabulated classifications and sorts them into ranked order based on frequency counts, (2) reconfiguration of classification criteria to generate disambiguated yes/no questions that function as selection and exclusion prompts, and (3) a selectable grid of TNM groupings that provides dynamic graphic demonstration of the effects of sequentially selecting or excluding specific classifications. Subjects were asked to evaluate the performance of this app after completing exercises simulating the staging of different types of cancers encountered during training.

**Results:**

Survey responses indicated high levels of agreement with statements supporting the usability and utility of this app. Subjects reported that its user interface provided a clear display with intuitive controls and that the exclusionary approach to TNM staging it demonstrated represented an efficient process of assignment that helped to clarify distinctions between tumor, node, and metastasis classifications. High overall usefulness ratings were bolstered by supplementary comments suggesting that this app might be readily adopted for use in clinical practice.

**Conclusions:**

A Web-based app that utilizes an exclusionary algorithm to prompt the assignment of tumor, node, and metastasis classifications may serve as an effective instructional aid demonstrating an efficient and informative approach to TNM staging.

## Introduction

The tumor/node/metastasis (TNM) staging system collaboratively developed and maintained by the American Joint Committee on Cancer and the International Union for Cancer Control plays a critical role in the evaluation and management of patients diagnosed with a range of different types of cancers [1]. Accurate staging based on assessment of the extent of anatomic spread of cancer at the time of diagnosis helps to determine prognosis based on correlated survival rates. Staging also helps to guide the planning of treatment, facilitates communication between providers working in different disciplines, and serves as the basis for identifying patients who may be eligible for enrollment in clinical trials [2].

Criteria for stage assignments have been established for 47 different types of cancers. Determination of a patient’s stage is based on the classification of three principal components that may be assessed at the point of diagnosis to determine a clinical stage, or after definitive surgery to determine a pathologic stage. Assignment of a tumor (T) classification ranging from T0-T4(a-d) is based on assessment of the size and extent of contiguous spread of the primary tumor. Assignment of a node (N) classification ranging from N0-N3(a-c) is based on assessment of the extent of spread of tumor to regional draining lymph nodes. Assignment of a metastasis (M) classification ranging from M0-M1(a-b) is based on assessment of the presence or absence of distant metastases (Table 1).

Additional prognostic factors that have proven to be significant in the staging of specific types of cancers include tumor grade, tumor location, mitotic rate, risk factors, histologic scores, and biochemical tumor marker levels. Compiled groupings of T, N, M, and prognostic factor classifications are sorted into tabular arrays that are stratified to define stages characterized as anatomic stages or prognostic groups ranging from 0-IV(A-C) in order of declining prognosis (Table 2).

**Table 1:** T, N, and M classifications for cancer of the lung.

Classification		Definition
**Primary tumor (T)**		
	TX	Primary tumor cannot be assessed
	T0	No evidence of primary tumor
	Tis	Carcinoma in situ
	T1	Tumor 3 cm or less in greatest dimension, surrounded by lung or visceral pleura, without bronchoscopic evidence of invasion more proximal than the lobar bronchus (ie, not in the main bronchus)
	T1a	Tumor 2 cm or less in greatest dimension
	T1b	Tumor more than 2 cm but 3 cm or less in greatest dimension
	T2	Tumor more than 3 cm but 7 cm or less or tumor with any of the following features (T2 tumors with these features are classified T2a if 5 cm or less): involves main bronchus, 2 cm or more distal to the carina; invades visceral pleura (PL1 or PL2); associated with atelectasis or obstructive pneumonitis that extends to the hilar region but does not involve the entire lung
	T2a	Tumor more than 3 cm but 5 cm or less in greatest dimension
	T2b	Tumor more than 5 cm but 7 cm or less in greatest dimension
	T3	Tumor more than 7 cm or one that directly invades any of the following: parietal pleural (PL3) chest wall (including superior sulcus tumors), diaphragm, phrenic nerve, mediastinal pleura, parietal pericardium; or tumor in the main bronchus less than 2 cm distal to the carina but without involvement of the carina; or associated atelectasis or obstructive pneumonitis of the entire lung or separate tumor nodule(s) in the same lobe
	T4	Tumor of any size that invades any of the following: mediastinum, heart, great vessels, trachea, recurrent laryngeal nerve, esophagus, vertebral body, carina, separate tumor nodule(s) in a different ipsilateral lobe
**Regional lymph nodes (N)**		
	NX	Regional nodes cannot be assessed
	N0	No regional lymph node metastasis
	N1	Metastasis in ipsilateral peribronchial and/or ipsilateral hilar lymph nodes and intrapulmonary nodes, including involvement by direct extension
	N2	Metastasis in ipsilateral mediastinal and/or subcarinal lymph node(s)
	N3	Metastasis in contralateral mediastinal, contralateral hilar, ipsilateral or contralateral scalene, or supraclavicular lymph node(s)
**Distant metastasis (M)**		
	M0	No distant metastasis
	M1	Distant metastasis
	M1a	Separate tumor nodule(s) in a contralateral lobe; tumor with pleural nodules or malignant pleural (or pericardial) effusion
	M1b	Distant metastasis

**Table 2:** Anatomic stage/prognostic groups for cancer of the lung.

Stage	T	N	M
Occult	Tx	N0	M0
0	Tis	N0	M0
IA	T1a	N0	M0
	T1b	N0	M0
IB	T2a	N0	M0
IIA	T2b	N0	M0
	T1a	N1	M0
	T1b	N1	M0
	T2a	N1	M0
IIB	T2b	N1	M0
	T3	N0	M0
IIIA	T1a	N2	M0
	T1b	N2	M0
	T2a	N2	M0
	T2b	N2	M0
	T3	N1	M0
	T3	N2	M0
	T4	N0	M0
	T4	N1	M0
IIIB	T1a	N3	M0
	T1b	N3	M0
	T2a	N3	M0
	T2b	N3	M0
	T3	N3	M0
	T4	N2	M0
	T4	N3	M0
IV	Any T	Any N	M1a
	Any T	Any N	M1b

The conventional approach to staging involves (1) selecting appropriate T, N, M, and prognostic factor classifications, (2) combining these classifications to generate a TNM grouping, and (3) locating this TNM grouping in the array of possible combinations to determine a corresponding stage. This combinatorial approach is inherently inefficient due to the fact that successive tumor and node classifications for different types of cancers are not always graded or mutually exclusive. Assignment of a T1 classification may be based on measurement of the diameter of the primary tumor, while assignment of a T2 classification may be based on identification of a pattern of local invasion. Assignment of an N2 classification may be based on confirmation of spread of tumor to a specific group of regional draining lymph nodes, while assignment of an N3 classification may be based on tabulation of the number of involved lymph nodes. As a result, accurate staging relies on scrupulous review of the criteria for each T, N, and prognostic factor classification to ensure that correct assignments are made.

An alternative approach to staging that seeks to optimize the efficiency of the process is predicated on the notion that unambiguous selection or exclusion of a T, N, M, or prognostic factor classification may serve to constrain the number of subsequent classifications that need to be reviewed to identify a correct TNM grouping. If a specific T, N, M, or prognostic factor classification can be selected based on review of its criteria, then any TNM groupings that do not include that classification can be excluded from further consideration. Alternatively, if a specific classification can be excluded without reservation, then any TNM groupings that include that classification can be excluded from further consideration. The set of TNM groupings that remain as viable options after a specific classification has been selected or excluded will most often encompass a restricted subset of classifications. In its elaboration, this exclusionary approach may effectively serve to minimize the number of classifications that need to be reviewed to accurately determine a patient’s stage.

## Methods

A Web-based app configured to demonstrate this approach to staging was developed as an instructional aid for trainees. A version coded in ActionScript 3.0 was iteratively adapted to incorporate the following key features [3]:

- an exclusionary algorithm that cycles through a sequence of (1) polling the set of TNM groupings listed for each anatomic stage to tabulate the number of times that each T, N, M, or prognostic factor classification is listed, (2) sorting the tabulated classifications in ascending order of frequency prioritized based on the extent of spread (M >N > T) and level of classification (N3 > N2 > N1> N0), (3) prompting selection or exclusion of the first ranked classification, and (4) excluding nullified TNM groupings from the set based on the response
- a grid of anatomic stage listings with corresponding TNM groupings incorporating selectable T, N, M, and prognostic factor classifications
- reconfiguration of the criteria specified for T, N, M, and prognostic factor classifications to generate yes/no questions phrased to (1) itemize the components of complex definitions, (2) disambiguate definitions that incorporate combined Boolean AND + OR conditions, and (3) minimize negative definitions (Table 3)

**Table 3:** Reconfiguration of classification criteria for cancer of the lung.

Classification	Definition	Yes/No question
T3: Complex definition	Tumor more than 7 cm or one that directly invades any of the following: parietal pleural (PL3) chest wall (including superior sulcus tumors), diaphragm, phrenic nerve, mediastinal pleura, parietal pericardium; or tumor in the main bronchus less than 2 cm distal to the carina but without involvement of the carina; or associated atelectasis or obstructive pneumonitis of the entire lung or separate tumor nodule(s) in the same lobe	Is the primary tumor >7 cm in greatest dimension? OR Does it invade any of these structures: Parietal pleura; Chest wall (including the superior sulcus); Diaphragm; Phrenic nerve; Mediastinal pleura; Parietal pericardium OR Does it involve the main bronchus at a site that is >2 cm distal to the carina without involvement of the carina? OR Is it associated with atelectasis or obstructive pneumonitis of the entire lung? OR Is there a separate tumor nodule in the same lobe?
T2: Combined Boolean AND + OR conditions	Tumor more than 3 cm but 7 cm or less or tumor with any of the following features (T2 tumors with these features are classified T2a if 5 cm or less): involves main bronchus, 2 cm or more distal to the carina; invades visceral pleura (PL1 or PL2); associated with atelectasis or obstructive pneumonitis that extends to the hilar region but does not involve the entire lung	Is the primary tumor >3 cm and ≤7 cm in greatest dimension? OR Is it associated with any of these findings:
		Involvement of the main bronchus at a site that is ≥2 cm distal to the carina; Invasion of the visceral pleura; Atelectasis or obstructive pneumonitis that extends to the hilar region but does not involve the entire lung
M0: Negative definition	No distant metastasis	Is there evidence of distant metastasis?

**Figure 1:**
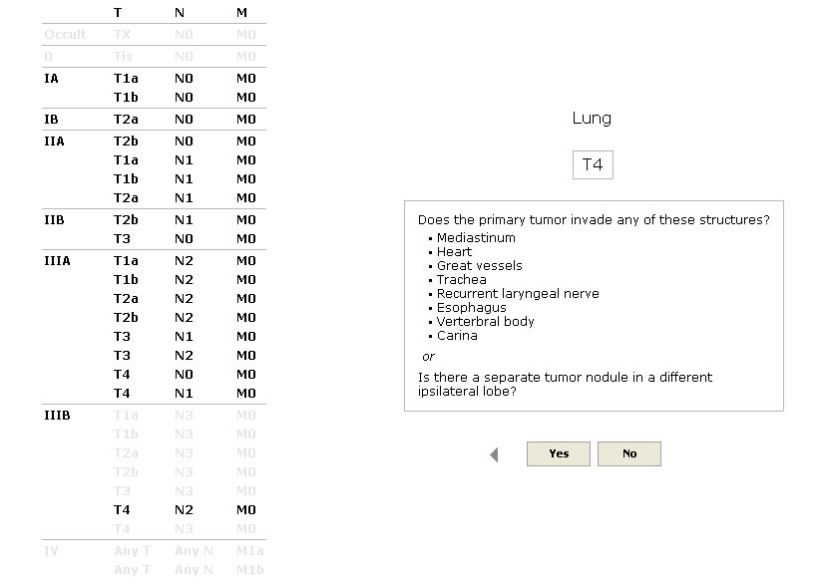
Clicking a response to the prompted question triggers fading of nullified TNM groupings. Forward and back arrows can be clicked to scroll through the sequence of selected and excluded classifications.

Users are prompted to select a tumor type to begin a staging exercise. Some tumor types require selection of secondary options which may include identification of a tumor subtype, anatomic location, age limit, or phase of staging (clinical vs pathologic). Selection of a tumor type shifts to a display that includes a grid of anatomic stage listings and a prompted yes/no question corresponding to the first ranked T, N, M, or prognostic factor classification (Figure 1).

Clicking a Yes or No button to answer the question will select or exclude the classification. If a specific classification is known at the point of entry, it can be directly selected by clicking on a corresponding entry in the grid of anatomic stage listings. Selection or exclusion of a classification triggers fading of nullified TNM groupings and cycling of the exclusionary algorithm. Subsequent prompted yes/no questions will appear in sequence until the set of TNM groupings has been narrowed to delimit a single TNM grouping and/or a specific anatomic stage. If a specific anatomic stage has been delimited with multiple TNM groupings that persist as viable options, a “Complete” button can be clicked to prompt further selection and exclusion. When a single TNM grouping has been identified, a terminal display lists the anatomic stage and T, N, M, and prognostic factor classifications with their corresponding criteria. Forward and back arrows can be clicked to scroll through the sequence of classifications with highlighting of selected answers and concordant enhancement and fading of associated TNM groupings.

### Recruitment

An evaluation study was conducted to assess the usability and utility of this app. Participating subjects recruited from regional clinical training programs by email solicitation included 14 internal medicine residents, 9 surgery residents, and 7 oncology fellows. Institutional review board approval was obtained prior to recruitment and enrollment. Subjects were provided with open access to a fully functional version of the app along with a set of basic operating instructions. They were asked to evaluate its performance during staging exercises that included assessment of a newly diagnosed cancer, restating of recurrent cancer, and review of an incorrectly staged cancer. Subjects were asked to complete these exercises with an eye towards assessment of how they might use this app to (1) simulate the staging of different types of cancers encountered during training and (2) study for board exams that test knowledge of TNM staging criteria. After completing the staging exercises, subjects were asked to complete a Web-based survey about the app focused on rating its ease of use, clarity of presentation, perceived accuracy, and potential for adoption in different clinical and educational settings. The survey consisted of 12 statements phrased to support the usability and utility of the app. Selectable responses were arrayed on a 5-point scale with options labeled “Strongly disagree”, “Disagree”, “Undecided”, “Agree”, and “Strongly agree”.

### Statistical Analysis

Median Likert scores and 25th and 75th quartiles were calculated for each survey item assigning a value of 1 to responses scored as “Strongly disagree”, 3 to items scored as “Undecided”, and 5 to items scored as “Strongly agree”. Subjects were also asked to rate the overall usefulness of the app on a scale ranging from 1 (“Not at all useful”) to 10 (“Essential”) and were provided with the option of entering free text comments about what they did or did not like about the app.

## Results

All 30 of the enrolled subjects completed the entire survey. Subjects expressed a high level of agreement with each of the 12 statements. No “Strongly disagree” responses were registered. The median level of agreement was “Agree” for 7 of 12 statements and “Strongly agree” for the remainder with minimal dispersion (Table 4).

**Table 4:** Survey responses.

Statement	Median	25-75 interquartile range
It was easy to learn how to use this app.	5	4-5
It is easy to navigate between different sections.	4	4-5
The information presented is clearly organized.	5	4-5
Presenting definitions in the form of Yes/No questions helps to clarify distinctions between different T, N, M, and prognostic factor classifications.	4	4-5
Breaking down complex definitions into sets of questions linked by and/or statements helps to clarify distinctions between different T, N, M, and prognostic factor classifications.	4	4-5
The graphic display helps to clarify distinctions between different anatomic stages.	4	4-5
The ability to review prior answers in sequence helps to clarify distinctions between different anatomic stages.	4	4-5
The approach to staging promoted by this app is efficient.	4.5	4-5
The anatomic stages assigned through use of this app are accurate and reliable.	4	4-5
This app would be a useful instructional aid to help prepare for board certification and re-certification exams.	4	3-5
This app would be a useful resource in clinical practice.	5	4-5
Providers who do not stage patients on a regular basis would be able to use this app without difficulty.	5	4-5

The median overall usefulness rating was 9 (25-75 interquartile range 8-9.75). Eighteen subjects elected to enter free text comments that ranged from general impressions of the app to specific criticisms of its navigability and functionality. While most of the general impressions were favorable (“Great application”, “Very useful clinically”), a few subjects expressed reservations about the limited scope of the app (eg, “It would be nice to have links to the appropriate staging guidelines or references”, “It would be great to see some of the hematologic malignancies like myeloma and lymphoma added”). The majority of the specific criticisms focused on problems that subjects experienced when trying to use the browser back button to navigate between screens. This problem is commonly encountered with the first use of platform-independent apps that run in browser plug-ins [4]. Internal navigation controls were moved to more intuitive locations in subsequent iterations as a result. A few of the subjects found staging exercises that began with prompted questions about the presence of distant metastases to be disconcerting at first, but on reflection they expressed an understanding of the logic of this approach.

## Discussion

### Principal Findings

Studies conducted to assess the validity of TNM staging after the most recent revision of specified criteria have demonstrated high levels of correlation between accurately assigned anatomic stages and overall survival for a range of different cancer types [5-14]. In light of the critical role that TNM staging has come to play in the treatment of cancer, it is curious that there do not appear to have been any published studies investigating the approaches that providers adopt to assign anatomic stages. Most of the studies evaluating TNM staging have focused on assessing rates of completion and accuracy of assignment without any examination of the process itself. Inventories of tumor registries have revealed that providers treating patients with specific types of cancers do not always assign anatomic stages or track the information needed to retrospectively confirm accurate assignments [15-17]. Studies that have compared assigned anatomic stages to adjudicated anatomic stages have shown that the accuracy of assignment may vary based on the expertise levels of providers and the specific types of cancers under consideration [18-21].

Resources that have been developed to assist providers engaged in the task of assigning anatomic stages include printed worksheets, encoded spreadsheets, wizards incorporated in electronic medical records, and an array of apps developed to run on smartphones and tablets [1,22-28]. While the controls and interfaces that they present vary to an extent, these resources universally implement combinatorial approaches to staging that rely on the selection of discrete T, N, M, and prognostic factor classifications to determine an anatomic stage. While the automated linkage of TNM groupings to anatomic stages provided by coded apps may ensure greater accuracy of assignment that obviates the need to refer to tabular arrays, users still need to review the criteria for each T, N, and prognostic group classification to ensure that correct groupings have been identified.

### Conclusion

This evaluation study demonstrated the perceived utility of a Web-based app configured to demonstrate an exclusionary approach to TNM staging. Subjects recruited from a pool of target users found that it was easy to use, and they deemed the approach to assignment that it employed to be informative, efficient, accurate, and reliable. It was interesting to note that while this app was originally developed as an educational resource, the statement that elicited the greatest number of “Disagree” responses focused on its potential use as an instructional aid to help prepare for board certification and re-certification exams. By way of contrast, statements suggesting that it could be used in clinical practice by providers with varying degrees of expertise elicited greater numbers of “Strongly agree” responses. This feedback may guide further development and investigation that may focus on evaluating the performance and acceptance of this app when it is deployed for use in simulated cancer staging exercises and real-time clinical practice.

## Abbreviations

TNM: tumour/node/metastasis
